# Reduction in opioid use with perioperative non-pharmacologic analgesia in total knee arthroplasty and ACL reconstruction: a systematic review

**DOI:** 10.1051/sicotj/2021063

**Published:** 2021-12-17

**Authors:** Ryan B Juncker, Faisal M Mirza, Joel J Gagnier

**Affiliations:** 1 Department of Orthopaedic Surgery, David Geffen School of Medicine, University of California, Los Angeles (UCLA) 615 Charles E Young Dr S, Rm. 410 Los Angeles CA 90095 USA; 2 Coastal Health Partners 65 Nielson St #102 Watsonville CA 95076 USA; 3 Department of Orthopaedic Surgery, Department of Epidemiology, University of Michigan 1500 E Medical Center Dr Ann Arbor MI 48109 USA

**Keywords:** Non-pharmacologic analgesia, Opioids, Cryoneurolysis, Percutaneous peripheral nerve stimulation, Auricular acupressure, Total knee arthroplasty, ACL reconstruction

## Abstract

*Introduction*: The world’s opioid epidemic has gotten increasingly severe over the last several decades and projects to continue worsening. Orthopedic surgery is the largest contributor to this epidemic, accounting for 8.8% of postoperative opioid dependence cases. Total knee arthroplasty (TKA) and anterior cruciate ligament (ACL) reconstruction are commonly performed orthopedic operations heavily reliant on opioids as the primary analgesic in the peri- and immediate postoperative period. These downfalls highlight the pressing need for an alternate, non-pharmacologic analgesic to reduce postoperative opioid use in orthopedic patients. The presented systematic review aimed to analyze and compare the most promising non-pharmacologic analgesic interventions in the available literature to guide future research in such a novel field. *Methods*: A systematic search of PubMed, MEDLINE, Embase, Cochrane, and Web of Science was performed for studies published before July 2020 based on the PRISMA (preferred reporting items for systematic reviews and meta-analyses) guidelines, and the obtained manuscripts were evaluated for inclusion or exclusion against strict, pre-determined criteria. Risk-of-bias and GRADE (grades of recommendation, assessment, development, and evaluation) assessments were then performed on all included studies. *Results*: Six studies were deemed fit for inclusion, investigating three non-pharmacologic analgesics: percutaneous peripheral nerve stimulation, cryoneurolysis, and auricular acupressure. All three successfully reduced postoperative opioid use while simultaneously maintaining the safety and efficacy of the procedure. *Discussion*: The results indicate that all three presented non-pharmacologic analgesic interventions are viable and warrant future research. That said, because of its slight advantages in postoperative pain control and operational outcomes, cryoneurolysis seems to be the most promising. Further research and eventual clinical implementation of these analgesics is not only warranted but should be a priority because of their vast potential to reduce orthopedics surgeries’ contribution to the opioid epidemic.

## Introduction

Total knee arthroplasty (TKA) and anterior cruciate ligament (ACL) reconstruction surgeries are two of the most performed orthopedic operations in the world [1–4]. TKA cases, revisions, and revisions per surgeon have all significantly increased in the last 10 years [2, 5]; ACL reconstruction has similarly become more prevalent over the last 30 years, particularly in the pediatric population, corresponding with the United States’ increase in youth sports injuries [1, 3, 6].

While both procedures are considered highly efficacious, with TKA having shown a 10- to 15-year implant survivorship rate of over 90% [2], and up to 92% of ACL reconstruction patients receiving “A” or “B” outcome scores in a 2018 systematic review by Sarraj et al. [6], they are both operations consistently associated with moderate to severe postoperative pain [1, 4]. Exposed by this are the sub-par anesthetic and analgesic perioperative pain management interventions used in the field of orthopedic surgery.

According to Li et al. 2019 review, a multimodal approach is currently considered best practice for TKA pain management [7, 8]. This approach subjects the patient to a wide variety of anesthetic and analgesic medications, including preemptive analgesia, neuraxial anesthesia, peripheral nerve blocks, patient controlled-analgesia, local infiltration analgesia, gabapentanoids, periarticular injections, non-steroidal anti-inflammatory drugs (NSAIDs), and opioids [7, 8]. A similar multimodal approach is currently used peri- and postoperatively for ACL reconstructions, highlighted by intra-articular injections, peripheral nerve blocks, regional anesthesia, oral gabapentanoids, and once again, the heavy use of NSAIDs and opioid medications [9–11].

The main downfall of both approaches is the heavy reliance on opioids, a strong and addictive class of painkillers associated with massive negative side effects [12]. Opioids present a high risk for misuse and abuse, with rates of misuse having been shown as up to 56.3% and addiction rates as high as 23.0% in prescribed users [12]. Rates of opioid-dependence disorders, non-medical opioid use, unauthorized distribution, and accidental overdoses have all skyrocketed in the United States over the past 25 years [13].

Additionally, the United States is in the middle of the world’s largest opioid epidemic [13–18], single-handedly consuming 80% of the world’s opioid supply [20]. More than 4% of the countries’ adult population currently misuses opioids [14], with 92% of these individuals misusing only physician-prescribed opioids and no other controlled substances [15], leading to an average of 130 Americans dying every day from opioid overdose [16]. Further, treatment of dependence, abuse, and overdose cases costs the United States economy up to $78.5 billion every year. Orthopedic surgery alone accounts for approximately 8.8% of postoperative opioid dependence cases in previously opioid-naive patients [19], prescribing more opioids than almost any other medical specialty [20].

The current multimodal analgesic approach mentioned above for both TKA and ACL reconstruction has attempted to combat this epidemic by lessening opioid use [7–11]; however, NSAIDs are the main alternative in these pain management strategies, which are also subject to severe negative consequences [21–23]. Harirforoosh et al. showed that NSAIDs could cause adverse effects in the gastrointestinal, cardiovascular, and renal systems [22] that limit or contraindicate NSAID use. NSAIDs have also been linked to postoperative bone and musculoskeletal soft tissue healing deficiencies, which is very concerning in orthopedic patients [21, 23].

The combination of the opioid epidemic and lack of viable alternative, long-term pharmacologic analgesics highlights the demanding need for a non-pharmacologic analgesic intervention in the perioperative period. An alternative non-pharmacologic analgesic working to the same efficacy as opioids and NSAIDs could, in theory, completely replace said drugs to manage pain in TKA and ACL reconstruction operations, ending orthopedic surgeries’ large contribution to the United States opioid crisis.

All of that said, non-pharmacologic perioperative analgesia is a very novel field of research, and the current literature is very limited. This systematic review intended to highlight and synthesize the available literature in the field, aiming to serve as a template for future research, effectively speeding up the process of establishing an efficacious non-pharmacologic analgesic for major knee surgery.

In doing so, the effectiveness of percutaneous peripheral nerve stimulation, percutaneous cyroneurolysis (percutaneous freezing of sensory nerves), and auricular acupressure were comparatively evaluated.

## Methods

### Systematic review methodology

A systematic search of the published literature was performed based on PRISMA (Preferred Reporting Items for Systematic Reviews and Meta-Analyses) guidelines for studies published before July 9th, 2020.

A Medical Subject Heading (MeSH) plus keyword search was performed across PubMed, MEDLINE, Embase, Cochrane, and Web of Science. The MEDLINE search was conducted as follows:

((Exp Electric Stimulation Therapy/ OR Exp Audioanalgesia/ OR (Nonpharmacologic* OR “non-pharmacologic*” OR Cryoanalges* OR cryoneuro* OR Neuromodulat* OR electrostimulat* OR “electric* stimulation” OR “electrical muscle stimulation” OR “interferential current” OR electroacupuncture OR “nerve stimulation” OR audioanalges* OR analgesi* OR (pain adj2 manag*)).ti,ab) AND ((Exp Arthroplasty, Replacement, Knee/ OR ((exp Knee/ OR exp Knee Joint/) AND (exp Arthroplasty/)) OR TKA.ti,ab OR (knee adj2 (replacement* OR arthroplast*)).ti,ab) OR ((Exp Anterior Cruciate Ligament Injuries/ OR exp anterior cruciate ligament/ OR ACL.ti,ab OR “anterior cruciate ligament”.ti,ab) AND (surgery.ti,ab OR reconstruction.ti,ab OR surgery.fs OR exp Surgical Procedures, Operative/)))) AND (perioperative.ti,ab OR “peri operative”.ti,ab OR (immediate* adj2 (postoperative OR “post operative”)).ti,ab OR exp Perioperative Care/ OR exp Perioperative Period/)

The articles obtained were then evaluated based on strict inclusion and exclusion criteria. Studies were included that focused on (1) analyzing the efficacy of a non-pharmacologic peri- or immediate postoperative intervention for TKA or ACL reconstruction operations, (2) analyzing the effect of said interventions on short and long-term postoperative opioid use, (3) analyzing the clinical and functional outcomes of procedures in patients treated with said interventions, and (4) used human patients.

Studies were excluded based on the following criteria: (1) focusing on operations other than TKA or ACL reconstruction; (2) focusing on patients who were previously diagnosed with opioid addiction; (3) analyzing the efficacy of a pharmacologic (drug-based) intervention; (4) analyzing the efficacy of a non-pharmacologic intervention used in the rehabilitation phase (after the immediate postoperative period).

All studies were screened independently based on the previously stated criteria. Articles were screened initially by title and then abstract, after which the full text of all remaining manuscripts was reviewed. All full-text manuscripts determined as meeting criteria were included for analysis.

### Outcomes of interest

The primary outcome of interest in the presented study was patients’ postoperative opioid use. Secondary outcomes of interest includes non-opioid postoperative medication use, pain scores, and clinical and functional surgical outcomes. These measures were intended to determine the ramifications of non-pharmacologic analgesia usage on the effectiveness of each operation.

### Quality of evidence

Each of the six included studies was also assessed for risk-of-bias: randomized controlled trials (RCTs) were assessed using the Revised Cochrane Risk-of-Bias Tool for Randomized Trials [24], case series were assessed using the Checklist for Quality Assessment of Non-Randomized Studies developed by Brazzelli et al. [25], and the retrospective chart review was assessed using Cochranes Tool to Assess Risk-of-Bias in Cohort Studies [26]. This assessment breakdown is represented visually in Tables 2–4. Overall potential risk-of-bias ratings for each study included low risk, high risk, or some concern [24].

Subsequently, the overall body of evidence was assessed using the GRADE (Grades of Recommendation, Assessment, Development, and Evaluation) criteria for each primary and secondary outcome. As such, the authors evaluated the evidence for each outcome based on study type, risk-of-bias, and the precision, consistency, and directness of the data. All GRADE assessment categories and outcomes evaluated can be seen in Table 5. The potential GRADE score for each outcome ranged from high quality to very low quality. Further, each outcome was rated as critical, important, or not important.

## Results

### Study selection

**Figure 1.**
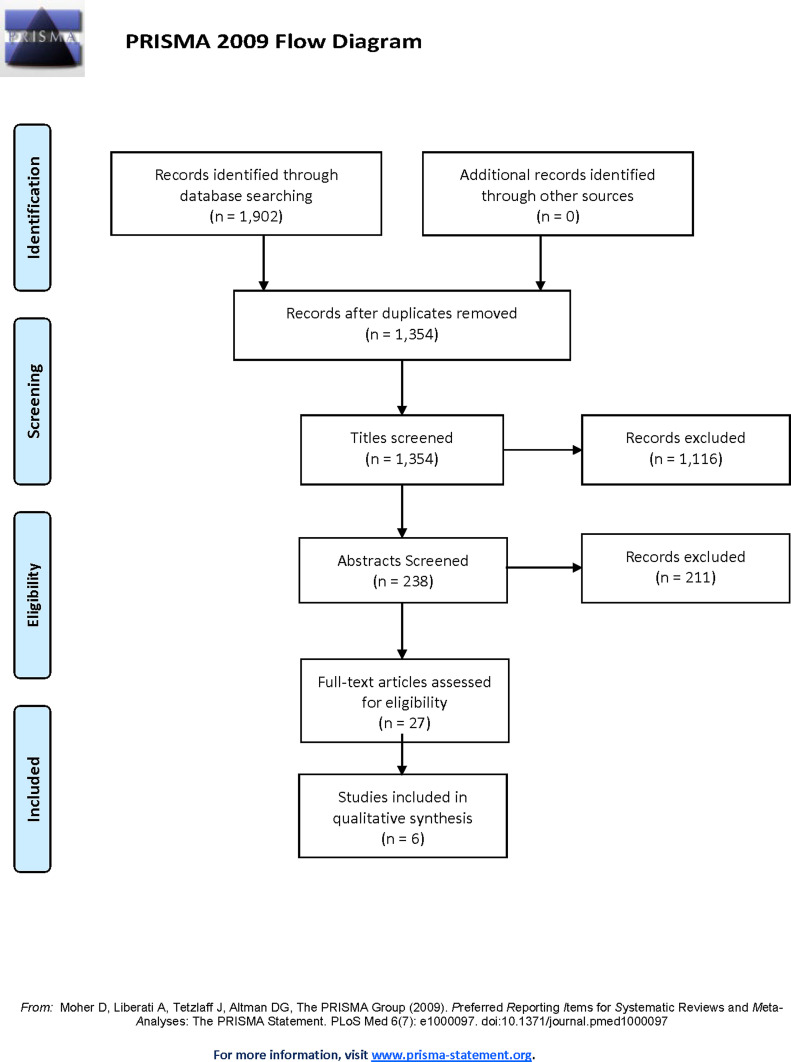
Preferred Reporting Items for Systematic Reviews and Meta-Analyses (PRISMA) flow diagram [42] illustrating the manuscript identification and screening process.

Database searches resulted in a total of 1902 publications to be screened. Initially, duplicates were removed, resulting in 1354 remaining papers. These papers were first screened by title – where 1116 papers were excluded, then abstract – where 211 more papers were excluded. Finally, full-text manuscripts of the remaining 27 publications were screened, and 6 were deemed to fit inclusion criteria. Figure 1 illustrates the screening process.

### Quality of evidence

The six included studies consisted of two RCTs [27, 28], three case series [29–31], and one retrospective chart review [32]. The risk-of-bias in each study were all determined to be either “low risk” or “some concern” (Tables 2–4).

GRADE quality of evidence was assessed for five separate outcomes: postoperative opioid use, other postoperative analgesic use, pain scores, clinical outcomes, and functional outcomes. The quality of evidence was rated as “low” for all, primarily due to the small sample sizes of most included studies, and all outcomes were graded as either “critical” or “important” (Table 5).

### Introduction to included studies: operations and non-pharmacologic analgesics investigated

Five included studies investigated the effects of non-pharmacologic analgesics on TKA patients [27, 29–32], while one studied ACL reconstruction patients [28]. Sample sizes ranged from a case series of 3 patients [31], to a 100-patient clinical trial [32]; details are shown in Table 1.

**Table 1.** Cumulative data for all studies fitting inclusion criteria.

Study	Non-pharmacologic modality	Number of patients (*N*)	Time period of intervention	Operation (TKA or ACL reconstruction)	Postoperative opioid use	Other postoperative analgesic use	Pain scores	Clinical outcomes	Functional outcomes
Ilfeld et al., 2019	Percutaneous peripheral nerve stimulation	7	Immediate post-op period (leads inserted by 20 h PO); continued until up to 6 weeks postoperatively	TKA	4/7 patients discontinued opioid use within the first week; median time to opioid cessaction = 6 days	–	Avg pain score during the first PO week was mild (<4/10) in 6/7 patients and continued to be so through PO week 4	WOMAC scores increased by avg of 46% by PO week 2 with 5/7 patients showing clinical improvement; 7/7 patients showed clinically significant improvement by PO week 12 with an avg improvement of 85%	6/7 patients completed the TUG walking test on day of hospital discharge; 5/7 patients reached preoperative level or better by PO week 2 for TUG walk test; 6/7 reached preoperative level or better on 6MWT by PO week 2; 6/7 showed >10% improvement on 6MWT compared to preoperative score by PO week 12
He et al., 2013	Auricular Acupressure	90 (45 treatment, 45 sham control)	Administered 4 times per day from POD 0–7	TKA	Treatment group used significantly less opioids delivered via PCA at all time points measured	–	No difference in PO pain intensity scores for the first 48 h after surgery; treatment group showed significantly lower PO pain intensity scores at PO days 3, 4, 5, and 7	Treatment group showed significantly better HSS scores at PO week 2; no difference was seen preoperatively or at 3 months PO	No ROM differences between groups
Dasa et al., 2016	Cryoneurolysis (percutaneous freezing of sensory nerves) using novel handheld device, “Iovera”	100 (50 treatment, 50 control)	Administered 5 days preoperatively and remained effective through the perioperative period	TKA	Treatment group showed significantly lower opioid use (45% less than control) for 12 weeks PO	–	Treatment group showed significantly lower PROMIS pain intensity scores at all measures PO	Treatment group showed significantly greater reductions in KOOS symptoms subscale scores from baseline at 6- and 12–week PO visits; treatment group showed significantly shorter PO LOS than control	–
Ilfeld et al. [30]	Percutaneous peripheral nerve stimulation	18 (case series); 10 series one, 8 series 2	Series 1: 6 patients tested one time <14 days after surgery; 4 patients tested >40 days after surgery; Series 2: beginning directly preoperatively, continuing up to 6 weeks PO	TKA	4/8 patients in series 2 discontinued opioid use within 1 week PO; Median time to opioid cessation was POD 16.5	–	Series 1: immediate pain relief of ≥ 50% reported in 9/10 patients, with average reduction of 75%; complete immediate pain relief was seen in 5/10 patients. Series 2: Mild (<4/10) average daily pain scores in majority of patients at rest, while walking and overall, during PO week 1; by PO week 12, 7/8 patients had well-controlled pain and 5/8 were pain-free	Series 2: By 6 weeks PO 8/8 patients had reached clinically significant improvement of WOMAC scores (at least 33% improvement), with an average improvement of 76%; Average improvement had increased to 86% by PO week 12	Series 2: By 2 weeks PO, 6/7 patients had reached preoperative level of 6MWT scores; by 12 weeks PO, 7/8 patients had improved scores by at least 10%, with an average improvement of 24%
Ilfield et al., 2017	Ultrasound-Guided Percutaneous Cryoneurolysis	3 (case series)	–	TKA	All 3 patients showed significantly less opioid use over a significantly decreased time period as compared to historical controls	–	All patients reported bring <2/10 on self-reported pain scale consistently for first 2–3 weeks PO	–	No gross motor deficit noted as compared to historical controls
Ilfeld et al., 2019	Ultrasound-Guided Percutaneous Peripheral Nerve Stimulation	10	Group 1 (5/10 patients) received stimulation for 5 min on POD 0, then sham stimulation for the subsequent 5 min, followed by constant stimulation for 30 min; Group 2 (5/10 patients) received sham stimulation for the initial 5 min on POD 0, then received stimulation for the next 5 min, then received constant stimulation for the following 30 min	ACL reconstruction	5/10 patients requested opioids after initial PO stimulation period	7/10 patients elected to initiate their canal nerve block for extra analgesia after initial PO stimulation period and before discharge; 8/10 patients used an optional perineural injection analgesic on POD 1 and 2, but only 3/10 patients used the same device on POD 3.	Both groups showed downward trend in pain scores over the initial 5 min of stimulation when tested every minute and a similar increase in pain scores over the 5-minute sham stimulation period. A mean pain score decrease of 84% was seen in both groups after 5 min of the secondary 30-minute stimulation period. Median pain score with movement was <2/10 by POD 4, and median pain score at rest across all patients was <4/10 on POD 1 and <2/10 by POD 3	–	No motor or sensory deficits reported in any patient

Three of these studies investigated the efficacy of percutaneous peripheral nerve stimulation, two percutaneous cryoneurolysis, and one auricular acupressure (Table 1). Lastly, the period of treatment and measurement ranged from five days preoperatively to six weeks postoperatively.

### Postoperative use of opioids and other pharmacologic analgesics

As seen in Table 1, five of the six included studies showed a significant decrease in opioid use for the treatment group, as compared to controls [27, 29–32]; the final study did not directly measure opioid consumption compared to control groups, so statistical significance could not be obtained. Even so, only 50% of patients in the final study undergoing peripheral nerve stimulation requested simultaneous opioids [28].

All three non-pharmacologic interventions proved successful at reducing postoperative opioid consumption. That said, the most overall success was seen in the Ilfeld et al. percutaneous peripheral nerve stimulation study, in which they showed a median time to opioid cessation of only six days [29].

Only Ilfeld et al. in 2018 reported on other pharmacologic analgesic medication usage, showing that 7/10 patients elected to turn on a canal nerve block to supplement non-pharmacologic treatment. However, the same paper also showed more encouraging data in that 8/10 patients elected to use an optional perineural injection analgesic on postoperative days (POD) 1 and 2, but only 3/10 patients still required the same treatment on POD 3 [28], suggesting peripheral nerve stimulation quickly became an independently successful analgesic intervention.

### Postoperative pain scores

All six included studies showed their respective interventions to be successful analgesics as measured by postoperative pain scores [27–32]. Only He et al. and Dasa et al. compared non-pharmacologic interventions to positive control groups, both reporting significantly lower pain scores for patients in the non-pharmacologic intervention condition [27, 32]. That said, Dasa et al. showed that cryoneurolysis treatment significantly decreased pain scores at all postoperative time points measured. Comparatively, He et al. auricular acupressure treatment was no more successful than the control for the first 48 h after surgery but reached statistical significance from day 3 onward, suggesting cryoneurolysis may be superior to auricular acupressure in the direct perioperative period [27, 32]. All other included studies reported mild pain scores for almost all treatment group patients (defined as <4/10 on a self-report pain scale) [28–31], the details of these results are outlined in Table 1.

### Clinical and functional outcomes

Clinical outcomes should be considered very important secondary outcomes for novel non-pharmacologic perioperative analgesics because it ensures that any successes with respect to reduced opioid consumption and pain management do not come at the expense of the primary operational goal. Four included studies reported data on the clinical outcome of each operation [27, 29, 30, 32]. As seen in Table 1, all four studies showed significant clinical improvement after surgery [27, 29, 30, 32]. While the variability in reporting mechanism makes direct comparisons between non-pharmacologic analgesic interventions difficult, consistency over time suggests that cryoneurolysis and percutaneous peripheral nerve stimulation are more successful than auricular acupressure in promoting positive clinical outcomes [27, 29, 30, 32], albeit to a small degree. Notably, none of the interventions negatively affect clinical outcomes [27, 29, 30, 32].

Functional outcomes of each operation were also analyzed as a secondary outcome. Five out of six studies reported such data [27–31]. As detailed in Table 1, all five studies (spanning auricular acupressure, cryoneurolysis, and percutaneous peripheral nerve stimulation) reported positive functional outcomes [27–31].

## Discussion

### Non-pharmacologic analgesic interventions reduce opioid use and manage peri- and postoperative pain

Cryoneurolysis, percutaneous peripheral nerve stimulation, and auricular acupressure all have the capability to successfully reduce postoperative opioid use in major knee surgery [27–32]. While small sample sizes make a direct comparison difficult, it appears that peripheral nerve stimulation may have a small primary advantage in reducing opioid consumption over the other two interventions. This interpretation is primarily based on the data (Table 1) showing that percutaneous peripheral nerve stimulation leads to a slim majority of patients (13/25 total) discontinuing opioid use within the first postoperative week or not requesting opioids at all [28–30]. The stated timeline is more drastic than any time to opioid cessation data from cryoneurolysis or auricular acupressure (though cryoneurolysis follows closely behind). However, the effect is almost certainly not significant, and there is a degree of variation within percutaneous peripheral nerve stimulation studies – one having shown a median time to opioid cessation of 6 days [29], and another 16.5 days [30]. Previous large-scale RCTs have shown the control group median time to opioid cessation in orthopedic surgery to range from 32 to 39 days postoperatively [33, 34], suggesting that the presented studies show large improvement from baseline levels. Moreover, it makes many patients who stopped taking opioids within the first week or forewent opioids completely more noteworthy.

Time to cessation of opioid use is an extremely important measure of non-pharmacologic analgesic success because opioid use of more than 31 days postoperatively is associated with a greater likelihood of chronic opioid use one year after surgery [35]. All non-pharmacologic analgesics assessed in the present study proved to be opioid-sparing [27–32]. Therefore, this data does not necessarily exhibit any superiority of results amongst the three modalities reviewed in this paper, but heavily emphasizes the advantages of utilizing any of these interventions over opioids.

Similarly, cryoneurolysis and percutaneous peripheral nerve stimulation reduced postoperative pain scores earlier in the perioperative period than did auricular acupressure [27–32]. Auricular acupressure reduced pain scores at POD 3 and beyond [27], but cryoneurolysis and percutaneous peripheral nerve stimulation did so immediately on POD 1 [28–32]. This early pain relief may have greater implications than just patient comfort, as higher pain on the day of surgery is a significant predictor of chronic, postoperative opioid use [36]. Thus, an even greater opioid reduction advantage may be seen in implementing cryoneurolysis or percutaneous peripheral nerve stimulation over auricular acupressure.

### Surgical outcomes

It is inherently important to ensure any novel analgesic does not negatively affect surgical outcomes. As such, it is of great significance that none of the presented non-pharmacologic interventions worsened clinical or functional outcomes following major knee surgery [27–32].

One major difference is noteworthy in that cryoneurolysis was the only modality to show improved clinical outcomes over a control group [32]; with significant improvements seen in the treatment group for KOOS Symptoms Subscale scores and postoperative length of stay [32]. Alternatively, percutaneous peripheral nerve stimulation and auricular acupressure only showed significant improvement in clinical or functional outcomes compared to preoperative baselines [27–30]. While such improvement certainly maintains the analgesic viability of percutaneous peripheral nerve stimulation and auricular acupressure, it simultaneously indicates a distinct advantage to cryoneurolysis. The identified gap in clinical outcomes can most likely be attributed to the robust ability of cryoneurolysis to decrease pain in the perioperative period [31, 32], avoiding uncontrolled pain, which leads to slower patient mobilization and a delayed start to rehabilitation, worsening clinical outcomes after TKA and ACL reconstruction [37, 38]. It has also been shown that preemptive analgesia can lessen the postoperative inflammatory response and perioperative pain [39, 40]. Fittingly, cryoneurolysis is the only of the three non-pharmacologic analgesics analyzed to be fully administered prior to surgery [29, 31].

### The most efficacious non-pharmacologic analgesic interventions

As alluded to above, cryoneurolysis, percutaneous peripheral nerve stimulation, and auricular acupressure appear to all be viable analgesic interventions for TKA and ACL reconstruction surgeries. That said, the presented data suggest that cryoneurolysis, closely followed by percutaneous peripheral nerve stimulation, maybe the most beneficial to implement in clinical settings.

Cryoneurolysis is the most successful intervention at inhibiting peri- and postoperative pain [31, 32]. Both cryoneurolysis and percutaneous peripheral nerve stimulation showed immediate postoperative analgesic efficacy [28–32], but cryoneurolysis separates as the most efficacious intervention because patients in such trials also exhibited slightly lower pain scores than those who underwent percutaneous peripheral nerve stimulation, while maintaining significant decreases in postoperative opioid use [31, 32]. Cryoneurolysis is also the only analyzed non-pharmacologic analgesic to improve clinical outcomes [32]. As detailed in Tables 2–5, cryoneurolysis also shows the strongest quality of evidence for included interventions. Combined with the factors described above, this leads to the conclusion that cryoneurolysis, followed in order by percutaneous peripheral nerve stimulation and auricular acupressure, is the most promising novel perioperative non-pharmacologic analgesic intervention for implementation into clinical use.

**Table 2.** ROB assessment for randomized controlled trials: Revised Cochrane ROB tool for Randomized Trials.

	He et al. 2013		Ilfeld et al. 2019	
Signaling Question	Comments	Response	Comments	Response
Domain 1: Risk of bias arising from the randomization process				
1.1 Was the allocation sequence random?	Random assignment with sealed envelope technique; sham controlled	Y	Randomized to treatment or sham treatment group using computer-generated lists and opaque envelopes.	Y
1.2 Was the allocation sequence concealed until participants were enrolled and assigned to interventions?	Sealed envelope technique used was utilized. The author specifically states that allocation remained concealed until after the data was analyzed	Y	Opaque envelopes were used.	Y
1.3 Did baseline differences between intervention groups suggest a problem with the randomization process?	No significant difference in baseline clinical tests, age or body weight between groups. Strict inclusion and exclusion criteria were utilized during the patient enrollment process.	N	Groups do not appear largely different in any way based on the demographic data provided; yet, no statistical analysis was run due to the small sample size of the present feasibility study, so it cannot be said with certainty that there are no statistically significant differences	PN
Risk-of-bias judgement: Domain 1		Low risk		Low risk
Domain 2: Risk of bias due to deviations from the intended intervention (effect of assignment to intervention)				
2.1. Were participants aware of their assigned intervention during the trial?	The study was sham-controlled, and there is no mention of participants having knowledge of their intervention group. However, the treatment was self-administered, so it is impossible to say with certainty that all participants were unaware they were performing a sham technique.	PN	The author specifically stated that participants were masked to their intervention throughout the trial.	N
2.2. Were carers and people delivering the interventions aware of participants assigned intervention during the trial?	The surgeons and experimenters were unaware of participants’ assigned interventions, but an acupuncturist had to train each participant to self-administer acupressure to either real or sham auricular acupressure points. The acupuncturist had no contact with the participants after training, and randomization was complete.	N	All experimenters and providers were masked to the participants intervention during the entirety of the trial.	N
2.3. If Y/PY/NI to 2.1 or 2.2: Were there deviations from the intended intervention that arose because of the trial context?		–		–
2.4 If Y/PY to 2.3: Were these deviations likely to have affected the outcome?		–		–
2.5. If Y/PY/NI to 2.4: Were these deviations from intended intervention balanced between groups?		–		–
2.6 Was an appropriate analysis used to estimate the effect of assignment to intervention?	The sham and experimental groups were statistically compared after randomization for sex, age, bodyweight, American Society of Anesthesiologists (ASA) status, and through Kellgren-Lawrence radiographic grading.	Y	Data was collected on participant demographics, health status, and success of lead implantation.	PY
2.7 If N/PN/NI to 2.6: Was there potential for a substantial impact (on the result) of the failure to analyze participants in the group to which they were randomized?		–		–
Risk-of-bias judgement: Domain 2		Low risk		Low risk
Domain 3: Missing outcome data				
3.1 Were data for this outcome available for all, or nearly all, participants randomized?	All 90 randomized patients completed the trial with eligible data.	Y	All 10 randomized patients completed the trial with eligible data. That said, 3 participants required or requested treatment slightly deviating from the original trial protocol. All discrepancies were clearly reported by the author.	PY
3.2 If N/PN/NI to 3.1: Is there evidence that the result was not biased by missing outcome data?		–		–
3.3 If N/PN to 3.2: Could missingness in the outcome depend on its true value?		–		–
3.4 If Y/PY/NI to 3.3: Is it likely that missingness in the outcome depended on its true value?		–		–
Risk-of-bias judgement: Domain 3		Low risk		Low risk
Domain 4: Risk of bias in measurement of the outcome				
4.1 Was the method of measuring the outcome inappropriate?	Outcomes were measured thoroughly and through multiple measures. Pain intensity scores (VAS scores), used dose and total dose of analgesic via PCA, incidence of adverse effects due to analgesic treatments, Hospital for Special Surgery (HSS) scores and range of motion measures were all used as an evaluation of the experimental non-pharmacologic analgesic treatment.	N	Multiple viable outcome measures were used to assess the efficacy of the presented percutaneous peripheral nerve stimulator as a non-pharmacologic analgesic. Pain level, opioid consumption, perceived sensory deficit, and whether or not the participant had used the perineural infusion analgesic were all recorded at multiple time points.	N
4.2 Could measurement or ascertainment of the outcome have differed between intervention groups?	All measures used were standardized and the sham-control group went through all of the same procedures as the experimental group.	N	All measures were standardized and experimenters, carers, and participant’s were all blinded to each participants intervention. Further, the sham group went through exactly the same procedure and measurements as the experimental group.	N
4.3 If N/PN/NI to 4.1 and 4.2: Were outcome assessors aware of the intervention received by study participants?	Participant intervention method remained blinded to the assessors until after all data was analyzed.	N	Participant intervention method remained double blinded until after data collection.	N
4.4 If Y/PY/NI to 4.3: Could assessment of the outcome have been influenced by knowledge of intervention received?		–		–
4.5 If Y/PY/NI to 4.4: Is it likely that assessment of the outcome was influenced by knowledge of intervention received?		–		–
Risk-of-bias judgement: Domain 4		Low risk		Low risk
Domain 5: Risk of bias in selection of the reported results				
5.1 Were the data that produced this result analyzed in accordance with a pre-specified analysis plan that was finalized before unblinded outcome data were available for analysis?	Number of participants needed for sufficient statistical power, dropout rates, and measures were discussed as if they were predetermined in the manuscript, but no protocol found.	PY	No statistical analysis was run because of the small sample size associated with it being a feasibility study.	NI
5.2 Is the numerical result being assessed likely to have been selected, on the basis of the results, from multiple eligible outcome measurements (e.g., scales, definitions, time points) within the outcome domain?	All outcome measures listed and tested were fully reported in the text and/or figures.	N	All outcome measures listed and tested were fully reported in the text and/or figures.	N
5.3 Is the numerical result being assessed likely to have been selected, on the basis of the results, from multiple eligible analyses of the data?	All listed measures in the methods are reported on and all data was analyzed before the researchers were unblinded to participants intervention type. The rating remains PN, however, as no statistical plan is listed or cited outside of the article itself.	PN	As no statistical analysis was run, it would not have been possible for the author to leave out certain representations of the statistical results.	N
Risk-of-bias judgement: Domain 5		Low risk	Rated as “some concern” because of NI response to 5.1.	Some Concern
Overall Risk of Bias				
Risk-of-bias judgement: All		Low risk	All but 1 domain is rated as low risk, but in accordance with the Cochrane risk-of-bias tool, the overall risk-of-bias is some concern.	Some Concern

*Possible responses to domain questions: Yes (Y), Probably Yes (PY), No (N), Probably No (PN), No Information (NI).

**Table 3.** ROB/quality assessment for case series.

	Ilfeld et al. [31]		Ilfeld et al. [28]		Ilfeld et al. [29]	
Criteria	Comments	Response	Comments	Response	Comments	Response
Were participants a representative sample selected from a relevant patient population (e.g., randomly selected from those seeking treatment despite age, duration of disease, primary of secondary disease and severity of disease)?	Patients undergoing TKA were offered novel analgesic treatment and those that consented to the treatment were included in the report.	Yes	The included participants fit inclusion criteria that well-represents the population of those who require TKA	Yes	All participants were patients previously scheduled for TKA at a single institution	Yes
Were the inclusion/exclusion criteria of participants clearly described?	Only statement in manuscript to this point is that patients undergoing TKA were offered the treatment; no statement of how many were offered or if there were limiting criteria	Unclear	Both are very clearly stated in the methods section	Yes	The only stated requirement was that participants must have been previously scheduled to undergo TKA at the authors home institution (University of California, San Diego)	Unclear
Were participants entering the study at a similar point in their disease progression (i.e., severity of disease)?	All participants were enrolled preoperatively	Yes	All participants were enrolled preoperatively	Yes	All participants were enrolled preoperatively	Yes
Was selection of patients consecutive?	Not stated	N/A	7 patients were enrolled and completed the study over a 7-month period, it was not stated how many TKA operations were performed by the surgeon over this duration or if all patients were offered enrollment into the study	Unclear	Not stated	N/A
Was data collection undertaken prospectively?	All data were collected in the postoperative period	Yes	All data were collected in the postoperative period; the study was prospectively registered	Yes	All data were collected in the postoperative period	Yes
Was the intervention (and comparison) clearly defined?	The intervention (ultrasound-guided percutaneous cyroneurolysis) was very clearly described and it was stated that the subjects were compared to historical controls (references for control studies listed)	Yes	The experimental intervention (percutaneous peripheral nerve stimulation) was clearly described; because of the small case series design of the study, no control group was used for comparison	Yes	The intervention (percutaneous peripheral nerve stimulation) was clearly described; participants were subjectively compared to historical controls because of the case series design	Yes
Was the intervention undertaken by someone experienced at performing the procedure?	It was not stated who performed the procedure	N/A	The performing surgeon was stated in the author contributions portion, and it was stated that the device used was FDA approved, but the surgeon’s experience is not stated	N/A	It is not stated who performed the procedure	N/A
Were the staff, place, and facilities where the patients were treated appropriate for performing the procedure (e.g., access to back-up facilities in hospital or special clinic)?	Procedures were performed at the University of California, San Diego hospital	Yes	Procedures were performed at the University of California, San Diego hospital	Yes	Procedures were performed at the University of California, San Diego hospital	Yes
Were objective (valid and reliable) outcome measures used, including satisfaction scale?	Pain scores, opioid use, analysis of motor function, and time of return to normal skin sensation were all used as outcome measures	Yes	Pain scores were measured at a multitude of time points through weekly clinic visits or phone calls to participants and daily diary keeping, functional recovery was assessed using the WOMAC index, TUG test and 6MWT	Yes	Pain scores, time to opioid cessation, the 6WMT and the WOMAC index were all used as outcome measures	Yes
Was follow-up long enough to detect important effects on outcomes of interest?	Participants were followed up with for at least 4 months postoperatively, seeming to be an adequate time to determine effects of an analgesic used in the perioperative/immediate postoperative period (moreover, all patients were followed until they had returned completely to normal feeling and function)	Yes	Outcomes were assessed through postoperative week 12 and the interventional treatment lasted for a maximum of 42 days (6 weeks), this allowed for adequate follow-up on functional recovery and chronic postoperative pain well after the intervention was completed.	Yes	Outcomes were assessed through postoperative week 12 and the interventional treatment lasted for a maximum of 6 weeks, this allowed adequate follow-up on functional recovery and chronic postoperative pain well after the intervention was completed.	Yes
Was information provided on non-respondents, dropouts?	Not stated, unclear if there were no dropouts or if the information was omitted because the study is a case series	No	Not stated, unclear if there were no dropouts or if the information was omitted because the study is a case series	No	Not stated, unclear if there were no dropouts or if the information was omitted because the study is a case series	No
Were the characteristics of withdrawals/dropouts similar to those that completed the study and therefore unlikely to cause bias?		N/A		N/A		N/A
Were the important prognostic factors identified (e.g., age, duration of disease, disease severity)?	Only parameter stated was that all participants were undergoing TKA	Unclear	Exclusion factors for the study encompassed many factors for increased risk of infection and other factors increasing surgical risk	Yes	The only parameters stated for enrollment were that participants be scheduled for TKA at the study site, it was not stated in the manuscript if other factors were collected	Unclear
Overall risk-of-bias judgment (High, Low, or Some Concern)	While no specific parts of the study design imply any greater risk-of-bias than all case series, the study is rated as “some concern” because no information was provided on dropouts or detailed enrollment parameters	Some Concern	This study was rated as some concern because the study design and inclusion/exclusion criteria were clear and pre-registered, but no information was provided on consecutive/inconsecutive participant enrollment, and no control group was used	Some Concern	While no specific parts of the study design imply any greater risk-of-bias than all case series, the study is rated as “some concern” because no information was provided on dropouts or detailed enrollment parameters	Some Concern

*Possible Responses: Yes, No, N/A, unclear.

**Table 4.** ROB assessment for retrospective chart review.

Dasa et al. [32]		
Signaling question	Comments	Response
Was selection of exposed and non‐exposed cohorts drawn from the same population?	The exposed and unexposed cohorts were drawn from the same site and were all consecutive patients; however, the 50 unexposed participants all received treatment directly before the 50 exposed participants	PY
Can we be confident in the assessment of exposure?	Cohorts were drawn from directly before and after the implementation of a new intervention protocol at the study site	DY
Can we be confident that the outcome of interest was not present at the start of the study?	The outcomes of interest are all postoperative measures	DY
Did the study match exposed and unexposed for all variables that are associated with the outcome of interest or did the statistical analysis adjust for these prognostic variables?	Yes, all variables associated with the outcome of interest were analyzed, and no statistically significant differences were found	DY
Can we be confident in the presence or absence of prognostic factors?	There were no statistically significant differences found in clinical/baseline outcome measures prior to intervention	DY
Can we be confident in the assessment of the outcome?	Yes, all measures used had been previously established as effective	DY
Was the follow-up of cohorts adequate?	Data was collected through the participants 3-month (12 weeks) follow-up clinic visits; the intervention was completed during the perioperative period	PY
Were co-interventions similar between groups?	It is clearly stated that all other anesthetic/analgesic interventions aside from the experimental intervention (percutaneous freezing of sensory nerves – cryoneurolysis) were identical between groups	DY

*Possible Responses: Definitely Yes (DY), Probably Yes (PY), Definitely No (DN).

**Table 5.** GRADE evidence profile.

Quality Assessment							Overall quality of evidence for outcome	Importance of outcome
Number of studies	Design	Limitations	Inconsistency	Indirectness	Imprecision	Other considerations		
Postoperative Opioid Use								
6	2 randomized controlled trials, 3 case series; 1 retrospective chart review	Some ROB concern for 1 RCT because no statistical analysis was run due to the small sample size; some ROB concern for the case series due to lacking information regarding participant enrollment and indirect control groups; no serious concerns for chart review	No serious inconsistencies	Some concern because the populations studied differed: RCTs vs. case series vs. retrospective chart review; measure was comparable across study	Small sample size (*n*) in 4/6 studies	None	Low	Critical
Other postoperative analgesic use								
1	Randomized controlled trial	Some ROB concerned because no statistical analysis was run due to small sample size	No serious inconsistencies	No serious indirectness concerns	Small sample size (*n*)	None	Low	Important
Pain scores								
6	2 randomized controlled trials, 3 case series; 1 retrospective chart review	Some ROB concern for 1 RCT because no statistical analysis was run due to the small sample size; some ROB concern for the case series due to lacking information regarding participant enrollment and indirect control groups; no serious concerns for chart review	No serious inconsistencies	Some concern because the populations studied differed: RCTs vs. case series vs. retrospective chart review; measure was comparable across study	Small sample size (*n*) in 4/6 studies	None	Low	Critical
Clinical outcomes								
4	1 randomized controlled trial, 2 case series, 1 retrospective chart review	No serious ROB concern for RCT; some ROB concern for the case series due to lacking information regarding participant enrollment and indirect control groups; no serious ROB concern for chart review	No serious inconsistencies	Some concern because the populations studied differed: RCTs vs. case series vs. retrospective chart review; measure was comparable across study	Small sample size (*n*) in 2/4 studies	None	Low	Critical
Functional Outcomes								
5	2 randomized controlled trials, 3 case series; 1 retrospective chart review	Some ROB concern for 1 RCT because no statistical analysis was run due to the small sample size; some ROB concern for the case series due to lacking information regarding participant enrollment and indirect control groups	No serious inconsistencies	Some concern because the populations studied differed: RCTs vs. case series; measure was comparable across study	Small sample size (*n*) in 4/5 studies	None	Low	Critical

*Possible answers for overall quality of evidence for outcome: high, moderate, low, very low.

*Possible answers for importance of outcome: critical, important, not important.

### Limitations of the presented studies

While considering all the presented data, it is also important to recognize the limitations of each study. Most limitations in the presented systematic review stem from the novelty of perioperative non-pharmacologic analgesic research. The largest overall limitation is the small number of suitable studies and small sample sizes investigated in said studies. As shown in Table 1, the two included studies were relatively large, with over 90 participants, but the remainder all had 18 or fewer. The small sizes led to other limitations, such as one study not having attempted to determine statistical significance [28], and two others having elected to statistically compare novel interventions to historical controls instead of a positive control group [30, 31]. Lastly, four of the six included studies were performed by the same first author [28–31]. While that fact limits the generalizability of the presented conclusions, the authors still deemed it apt to include all four of the studies for two reasons: (1) given the novelty of the field and a small amount of available literature, these studies also make up much of the literature regarding perioperative non-pharmacologic analgesics, and (2) all four of the studies met the systematic review’s inclusion criteria.

### Implications of the data

As highlighted in the Introduction section, the primary benefit of a successful perioperative non-pharmacologic analgesic would be to replace opioids as the primary postoperative analgesic in major knee surgery. Doing so would eliminate the vast negative effects of opioid use, including concerningly high misuse and abuse rates in prescribed users and a high frequency of adverse side effects [12]. Moreover, this is of particular importance to orthopedic researchers as the United States is currently in the middle of the world’s worst opioid epidemic [13–18], with opioid addiction and overdose rates rising rapidly over the last quarter-century and orthopedic surgery being the single largest prescribing medical specialty [19, 20]. Fortunately, the data presented in this systematic review indicates that several non-pharmacologic interventions may be viable to replace opioids as the primary perioperative analgesic for TKA and ACL reconstruction operations.

### Direction of future research

Future research into perioperative non-pharmacologic analgesics is certainly warranted. The first and most telling research that should be performed is to implement full-scale, multi-site RCTs for cryoneurolysis and percutaneous peripheral nerve stimulation. While the data from pilot studies are very promising, RCTs would drastically improve the reliability of results seen and overcome the available literature’s current issue with small sample sizes. Moreover, it would be very telling to measure longer-term follow-ups, allowing researchers to determine if there are any negative long-term effects on nerve function from either cryoneurolysis or percutaneous peripheral nerve stimulation. Although studies utilizing cryoneurolysis to treat chronic pain have shown it to be safe and reversible over time [41], so completing these long-term studies may not be as pressing.

Other interesting extensions of the presented studies would be determining if an additive or synergistic effect can be seen by simultaneously implementing auricular acupressure and either cryoneurolysis or percutaneous peripheral nerve stimulation. Lastly, a notable distinction exists in the presented papers evaluating cryoneurolysis and percutaneous peripheral nerve stimulation that some utilize ultrasound guidance for implementation, and some do not. While it seems reasonable that ultrasound guidance is safer and allows for more detailed lead placement, similar results were seen in the presented review for studies including or excluding ultrasound guidance. Hence, a trial directly comparing outcomes for these groups would be important in setting clinical best practices.

## Conclusion

The present systematic review aimed to analyze and compare the available literature on perioperative non-pharmacologic analgesic interventions in the hopes of advancing a novel field of orthopedic research, with the end goal of lessening postoperative opioid use after TKA and ACL reconstruction operations. As such, the authors conclude that cryoneurolysis, percutaneous peripheral nerve stimulation, and auricular acupressure all appear to be viable and successful non-pharmacologic analgesics in the perioperative period. Of the three, cryoneurolysis seems the most promising moving into future research.

## Conflict of interest

R. Juncker: The author declares that they have no relevant financial or non-financial interests to report.

F. Mirza: The author declares that they have no relevant financial or non-financial interests to report.

J. Gagnier: The author declares that they have no relevant financial or non-financial interests to report.

## Funding

This research did not receive any specific funding.

## Ethical approval

Ethical approval was not required.

## Informed consent

This article does not contain any studies performed by the authors involving human subjects.

## Author contributions

R. Juncker: *Conceptualization, Methodology, Investigation, Writing original draft, Reviewing and Editing*; F. Mirza: *Conceptualization, Methodology, Investigation, Reviewing and Editing;* J. Gagnier: *Conceptualization, Methodology, Investigation, Reviewing and Editing*

## References

[1] Siegel L, Vandenakker-Albanese C, Siegel D (2012) Anterior cruciate ligament injuries: anatomy, physiology, biomechanics, and management. Clin J Sport Med 22(4), 349–355. 22695402 10.1097/JSM.0b013e3182580cd0

[2] Delanois RE, Mistry JB, Gwam CU, Mohamed NS, Choksi US, Mont MA (2017) Current epidemiology of revision total knee arthroplasty in the United States. J Arthroplasty 32(9), 2663–2668. 28456561 10.1016/j.arth.2017.03.066

[3] Mall NA, Chalmers PN, Moric M, Tanaka MJ, Cole BJ, Bach BR Jr, Paletta GA Jr (2014) Incidence and trends of anterior cruciate ligament reconstruction in the United States. Am J Sports Med 42(10), 2363–2370. 25086064 10.1177/0363546514542796

[4] Hasegawa M, Tone S, Naito Y, Wakabayashi H, Sudo A (2019) Prevalence of persistent pain after total knee arthroplasty and the impact of neuropathic pain. J Knee Surg 32(10), 1020–1023. 30414165 10.1055/s-0038-1675415

[5] Bozic KJ, Kamath AF, Ong K, Lau E, Kurtz S, Chan V, Vail TP, Rubash H, Berry DJ (2015) Comparative epidemiology of revision arthroplasty: failed THA poses greater clinical and economic burdens than failed TKA. Clin Orthop Relat Res 473(6), 2131–2138. 25467789 10.1007/s11999-014-4078-8 PMC4418985

[6] Sarraj M, de Sa D, Shanmugaraj A, Musahl V, Lesniak BP (2019) Over-the-top ACL reconstruction yields comparable outcomes to traditional ACL reconstruction in primary and revision settings: a systematic review. Knee Surg Sports Traumatol Arthrosc 27(2), 427–444. 30078121 10.1007/s00167-018-5084-2

[7] Li JW, Ma YS, Xiao LK (2019) Postoperative pain management in total knee arthroplasty. Orthop Surg 11(5), 755–761. 31663286 10.1111/os.12535 PMC6819170

[8] Elmallah RK, Chughtai M, Khlopas A, Newman JM, Stearns KL, Roche M, Kelly MA, Harwin SF, Mont MA (2018) Pain control in total knee arthroplasty. J Knee Surg 31(6), 504–513. 28719941 10.1055/s-0037-1604152

[9] Secrist ES, Freedman KB, Ciccotti MG, Mazur DW, Hammoud S (2016) Pain management after outpatient anterior cruciate ligament reconstruction: a systematic review of Randomized Controlled Trials. Am J Sports Med 44(9), 2435–2447. 26684664 10.1177/0363546515617737

[10] Abdallah FW, Brull R, Joshi GP (2019) Pain management for ambulatory arthroscopic anterior cruciate ligament reconstruction: evidence-based recommendations from the Society for Ambulatory Anesthesia. Anesth Analg 128(4), 631–640. 30649069 10.1213/ANE.0000000000003976

[11] Rao AG, Chan PH, Prentice HA, Paxton EW, Funahashi TT, Maletis GB (2019) Risk factors for opioid use after anterior cruciate ligament reconstruction. Am J Sports Med 47(9), 2130–2137. 31303011 10.1177/0363546519854754

[12] Vowles KE, McEntee ML, Julnes PS, Frohe T, Ney JP, van der Goes DN (2015) Rates of opioid misuse, abuse, and addiction in chronic pain: a systematic review and data synthesis. Pain 156(4), 569–576. 25785523 10.1097/01.j.pain.0000460357.01998.f1

[13] Stoicea N, Costa A, Periel L, Uribe A, Weaver T, Bergese SD (2019) Current perspectives on the opioid crisis in the US Healthcare System: a comprehensive literature review. Medicine (Baltimore) 98(20), e15425. 31096439 10.1097/MD.0000000000015425 PMC6531094

[14] Skolnick P (2018) The opioid epidemic: crisis and solutions. Annu Rev Pharmacol Toxicol 58, 143–159. 28968188 10.1146/annurev-pharmtox-010617-052534

[15] Lyden J, Binswanger IA (2019) The United States opioid epidemic. *Semin Perinatol* 43(3), 123–131. 30711195 10.1053/j.semperi.2019.01.001 PMC6578581

[16] Reider B (2019) Opioid Epidemic. Am J Sports Med 47(5), 1039–1042. 30943075 10.1177/0363546519836727

[17] Wilkerson RG, Kim HK, Windsor TA, Mareiniss DP (2016) The opioid epidemic in the United States. *Emerg Med Clin North Am* 34(2), e1–e23. 27133253 10.1016/j.emc.2015.11.002

[18] Shipton EA, Shipton EE, Shipton AJ (2018) A review of the opioid epidemic: What do we do about it? Pain Ther 7(1), 23–36. 29623667 10.1007/s40122-018-0096-7 PMC5993689

[19] Trasolini NA, McKnight BM, Dorr LD (2018) The opioid crisis and the orthopedic surgeon. *J Arthroplasty* 33(11), 3379–82.e1. 30075877 10.1016/j.arth.2018.07.002

[20] Leopold SS, Beadling L (2017) Editorial: The opioid epidemic and orthopaedic surgery-no pain, Who gains? Clin Orthop Relat Res 475(10), 2351–2354. 28762150 10.1007/s11999-017-5454-y PMC5599414

[21] Ghosh N, Kolade OO, Shontz E, Rosenthal Y, Zuckerman JD, Bosco JA 3rd, Virk MS (2019) Nonsteroidal Anti-Inflammatory Drugs (NSAIDs) and their effect on musculoskeletal soft-tissue healing: a scoping review. *JBJS Rev* 7(12), e4. 10.2106/JBJS.RVW.19.00055 31851037

[22] Harirforoosh S, Asghar W, Jamali F (2013) Adverse effects of nonsteroidal antiinflammatory drugs: an update of gastrointestinal, cardiovascular and renal complications. J Pharm Sci 16(5), 821–847. 10.18433/j3vw2f 24393558

[23] Lisowska B, Kosson D, Domaracka K (2018) Positives and negatives of nonsteroidal anti-inflammatory drugs in bone healing: the effects of these drugs on bone repair. Drug Des Devel Ther 12, 1809–1814. 10.2147/DDDT.S164565 PMC6016595 29950815

[24] Cochrane Bias RoB 2: A Revised Cochrane Risk-of-Bias Tool for Randomized Trials. https://methods.cochrane.org/bias/resources/rob-2-revised-cochrane-risk-bias-tool-randomized-trials

[25] Brazzelli M, Cruickshank M, Tassie E, et al. (2015) Collagenase clostridium histolyticum for the treatment of Dupuytren’s contracture: systematic review and economic evaluation. NIHR Journals Library: Southampton (UK). (Health Technology Assessment, No. 19.90.) Appendix 5, Risk-of-bias checklist: case series. Available from: https://www.ncbi.nlm.nih.gov/books/NBK326583/10.3310/hta19900 PMC4781188 26524616

[26] Cochrane. Tool to Assess Risk of Bias in Cohort Studies. https://methods.cochrane.org/bias/sites/methods.cochrane.org.bias/files/public/uploads/Tool%20to%20Assess%20Risk%20of%20Bias%20in%20Cohort%20Studies.pdf.

[27] He BJ, Tong PJ, Li J, Jing HT, Yao XM (2013) Auricular acupressure for analgesia in perioperative period of total knee arthroplasty. Pain Med 14(10), 1608–1613. 23865512 10.1111/pme.12197

[28] Ilfeld BM, Said ET, Finneran JJ IV, et al. (2019) Ultrasound-guided percutaneous peripheral nerve stimulation: neuromodulation of the femoral nerve for postoperative analgesia following ambulatory anterior cruciate ligament reconstruction: a proof of concept study. Neuromodulation 22(5), 621–629. 30160335 10.1111/ner.12851 PMC6767389

[29] Ilfeld BM, Ball ST, Gabriel RA, et al. (2019) A feasibility study of percutaneous peripheral nerve stimulation for the treatment of postoperative pain following total knee arthroplasty. Neuromodulation 22(5), 653–660. 30024078 10.1111/ner.12790 PMC6339601

[30] Ilfeld BM, Ball ST, Cohen SP, Hanling SR, Fowler IM, Wongsarnpigoon A, Boggs JW (2019) Percutaneous peripheral nerve stimulation to control postoperative pain, decrease opioid use, and accelerate functional recovery following orthopedic trauma. Mil Med 184(Suppl 1), 557–564. 30901395 10.1093/milmed/usy378 PMC6523432

[31] Ilfeld BM, Gabriel RA, Trescot AM (2017) Ultrasound-guided percutaneous cryoneurolysis providing postoperative analgesia lasting many weeks following a single administration: a replacement for continuous peripheral nerve blocks? A case report. Korean J Anesthesiol 70(5), 567–570. 29046778 10.4097/kjae.2017.70.5.567 PMC5645591

[32] Dasa V, Lensing G, Parsons M, Harris J, Volaufova J, Bliss R (2016) Percutaneous freezing of sensory nerves prior to total knee arthroplasty. Knee 23(3), 523–528. 26875052 10.1016/j.knee.2016.01.011

[33] Hah JM, Cramer E, Hilmoe H, et al. (2019) Factors associated with acute pain estimation, postoperative pain resolution, opioid cessation, and recovery: secondary analysis of a Randomized Clinical Trial. JAMA Netw Open 2(3), e190168. 30821824 10.1001/jamanetworkopen.2019.0168 PMC6484627

[34] Hah J, Mackey SC, Schmidt P, et al. (2018) Effect of perioperative gabapentin on postoperative pain resolution and opioid cessation in a mixed surgical cohort: a Randomized Clinical Trial. JAMA Surg 153(4), 303–311. 29238824 10.1001/jamasurg.2017.4915 PMC5933381

[35] Hills JM, Carlile CR, Archer KR, et al. (2020) Duration and dosage of opioids after spine surgery: implications on outcomes at 1 Year. Spine (Phila Pa 1976) 45(15), 1081–1088. 32675616 10.1097/BRS.0000000000003446

[36] Goesling J, Moser SE, Zaidi B, Hassett AL, Hilliard P, Hallstrom B, Clauw DJ, Brummett CM (2016) Trends and predictors of opioid use after total knee and total hip arthroplasty. Pain 157(6), 1259–1265. 26871536 10.1097/j.pain.0000000000000516 PMC4868627

[37] Rutherford RW, Jennings JM, Dennis DA. 2017. Enhancing recovery after total knee arthroplasty. Orthop Clin North Am 48(4), 391–400. 28870300 10.1016/j.ocl.2017.05.002

[38] Łyp M, Stanisławska I, Witek B, Majerowska M, Czarny-Działak M, Włostowska E (2018) The timing of rehabilitation commencement after reconstruction of the anterior cruciate ligament. Adv Exp Med Biol 1096, 53–57. 29737496 10.1007/5584_2018_210

[39] Wall PD (1988) The prevention of postoperative pain. Pain 33(3), 289–290. 3419835 10.1016/0304-3959(88)90286-2

[40] Scott WN (2011) Insall & Scott Surgery of the Knee E-Book. Elsevier Health Sciences.

[41] Smiley A, McGuire J (2018) Cryoneurolysis for the treatment of sensory nerve pain. AANA J 86(6), 495–503. 31584424

[42] Moher D, Liberati A, Tetzlaff J, Altman DG, The PRISMA Group (2009) Preferred reporting items for systematic reviews and meta-analyses: the PRISMA statement. PLoS Med 6(7), e1000097. 19621072 10.1371/journal.pmed.1000097 PMC2707599

